# Nitric oxide synthase and polycystic kidney disease

**Published:** 2015-06-20

**Authors:** Viroj Wiwanitkit

**Affiliations:** Surin Rajabhat University, Surin, Thailand

**Keywords:** Nitric oxide synthase, Polycystic kidney disease, Chronic kidney disease

**Implication for health policy/practice/research/medical education:**

Endothelial nitric oxide plays a crucial role in the control of local hemodynamics and systemic blood pressure. Thus, it has been proposed that gene coding for endothelial nitric oxide synthase (eNOS) could have a modifying effect on hypertension and related complications in autosomal dominant polycystic kidney disease (ADPKD). As endothelial dysfunction and oxidative stress are evident early in ADPKD patients, eNOS holds therapeutic promise in the treatment of ADPKD.

Polycystic kidney disease (PKD) is a multisystem disorder characterized by the formation of cysts within the kidneys. PKD is mainly caused by the unfolding of its immunoglobulin-like PKD domains. There are two major forms of PKD; autosomal dominant (ADPKD) that caused by mutations of PKD1 and PKD2 (1) and autosomal recessive (ARPKD) caused by mutations in PKHD1 gene (2,3). Although, cellular changes and mechanisms involved in initial stages of cyst formation might differ between ADPKD and ARPKD, both are important causes of kidney failure, morbidity and mortality in children and adults of all racial groups worldwide (4). Further, it is clear that the disease progression in the ADPKD and ARPKD involves almost similar pathogenetic mechanisms (5,6). Hypertension is an important factor in the progression of kidney failure ADPKD patients, 60% of ADPKD patients exhibit high blood pressure even before the impairment of kidney function (7).

Accumulating evidence, including findings of elevation of arterial blood pressure in mice lacking the endothelial nitric oxide synthase (eNOS) gene, strongly suggests that alteration in NO metabolism is implicated in hypertension (8). Nitric oxide synthases (NOSs) catalyze the conversion of L-arginine into l-citrulline and nitric oxide (9,10). The gene coding for endothelial NOS (NOS3) is located on chromosome 7q35-36 (11) and harboring several polymorphic variants that can alter nitric oxide synthesis. These include, promoter polymorphism (-786T > C), Glu298Asp is a missense variant in exon 7 and 27-base pair (bp) variable number of tandem repeat in intron-4. The -786 C allele of promoter polymorphism acts as a repressor of NOS3 transcription (12). The Glu298Asp and intron-4 VNTR are also known to alter endothelial NOS expression (11,13). Various studies have shown that nitric oxide synthase gene polymorphisms in PKD. A recent report on “nitric oxide synthase VNTR (intron 4 a/b) polymorphism and PKD” is very interesting (14). Elumalai et al studied the clinical relationship between VNTR genetic polymorphism and clinical course in patients with ADPKD (14). Elumalai et al showed the “significant association between the 27-bp VNTR and chronic kidney disease (CKD) advancement among the ADPKD patients in the South Indian population (14). The other study by analyzing four tagging SNPs and two more well studied polymorphisms (Intron 4 VNTR and Glu298Asp) of the NOS3 gene investigated to identify the potential modifier effect of NOS3 gene on the progression of CKD in ADPKD (15). However, they did not find any evidence for the involvement of NOS3 tag-SNPs in the progression to CKD in ADPKD patients (15). Direct analysis of NOS3 gene polymorphisms in ADPKD patients has also revealed inconclusive results from many populations (Table 1).

**Table 1:** Studied related to NOS3 gene polymorphisms and ADPKD in different regions of the world

**Country**	**Association**	**Reference**
-786T > C		
Belgium	No	(16)
Spain	Yes	(17)
Iran	No	(18)
Glu298Asp		
Japan	Yes	(19)
Japan	Yes	(20)
Belgium	Yes	(16)
United kingdom	No	(21)
Czech Republic	No	(22)
Spain	Yes	(23)
Spain	Yes	(17)
Greece	Yes	(24)
Iran	No	(18)
India	No	(15)
Intron-4 27bp VNTR		
Czech Republic	Yes	(25)
Belgium	No	(16)
Japan	No	(20)
Greece	Yes	(26)
India	Yes	(14)
India	No	(15)

Multiple studies have shown that nitric oxide negatively regulates the renin-angiotensin aldosterone system (RAAS) by inhibiting ACE activity and angiotensin II type 1 receptors (27). Use of angiotensin converting enzyme inhibitors (ACE inhibitors) and angiotensin receptor blockers (ARBs) in controlling the CKD progression is further supporting the role of endothelial nitric oxide synthase.

Evidence suggests that endothelial nitric oxide synthase NOS3) is post-translationally lysine acetylated, leading to increased NO production in the endothelium. Expression of NOS3 is not only controlled by gene polymorphisms but also by its HDAC-dependent posttranscriptional regulation (28). Endothelial dysfunction and oxidative stress are evident early in ADPKD patients, even in those with preserved kidney function. Hence, HDAC1-mediated deacetylation of NOS3 may represent a novel target for endothelial dysfunction in ADPKD. Trichostatin A (TSA), a histone deacetylase inhibitor, was found to inhibit cyst formation in pkd2-deficient zebrafish and Pkd2^–/–^ mouse embryos (29,30). This paper provides a solid base for eNOS role of in the development of hypertension induced CKD in ADPKD patients.

## Author’s contribution

VW is the single author of the manuscript.

## Conflicts of interest

The author declared no competing interests.

## Ethical considerations

Ethical issues (including plagiarism, data fabrication, double publication) have been completely observed by the author.

## Funding/Support

None.
